# Synchronous lung adenocarcinoma and pulmonary carcinoid tumorlets in different lobes: a case report

**DOI:** 10.3389/fonc.2026.1972191

**Published:** 2026-09-29

**Authors:** Yan-Qing Li, Yan-Zhao Xu

**Affiliations:** Department of Thoracic Surgery, Fourth Hospital of Hebei Medical University, Shijiazhuang, China

**Keywords:** case report, lung adenocarcinoma, multiple pulmonary nodules, pulmonary carcinoid tumorlet, pulmonary neuroendocrine cell proliferation

## Abstract

**Background:**

Pulmonary carcinoid tumorlets are small (< 5 mm) neuroendocrine proliferations that are usually identified incidentally in resected lung tissue. Their coexistence with a spatially separate lung adenocarcinoma is uncommon and may complicate the interpretation of multiple pulmonary nodules.

**Case summary:**

A 71-year-old woman with multiple bilateral pulmonary micronodules that had remained radiologically stable for approximately 2 years was admitted after a newly detected lesion in the apicoposterior segment of the left upper lobe was considered suspicious for lung cancer. She reported occasional chest tightness and intermittent cough with sputum and had never smoked. Bronchoscopy and brush cytology were negative. The patient underwent video-assisted thoracoscopic wedge resection of the left upper lobe lesion. Frozen-section examination showed adenocarcinoma, prompting a left upper lobectomy with systematic lymph-node dissection. A left lower lobe wedge resection was performed to sample representative micronodules. Final pathology showed a 3.0-cm invasive adenocarcinoma (approximately 80% acinar and 20% lepidic patterns), without visceral pleural, definite vascular invasion, or metastasis in 17 sampled lymph nodes (pT1cN0M0, stage IA3). The left lower lobe nodules were reported as pulmonary carcinoid tumorlets and were positive for AE1/AE3, synaptophysin, chromogranin A, CD56, and TTF-1, with a Ki-67 index of approximately 2%. The adenocarcinoma harbored an *EGFR* exon 21 L858R mutation. She was discharged 1 week after surgery.

**Conclusion:**

This case illustrates that pulmonary micronodules accompanying a dominant lung cancer may represent a separate neuroendocrine proliferation rather than intrapulmonary metastases. Diagnosis requires correlation of lesion size, morphology, and immunophenotype. Unsampled contralateral nodules could remain radiologically indeterminate and undergo structured surveillance.

## Introduction

Lung adenocarcinoma is the most common histologic subtype of primary lung cancer (1, 2). Pulmonary neuroendocrine proliferations encompass pulmonary neuroendocrine cell hyperplasia, carcinoid tumorlets, and carcinoid tumors. A carcinoid tumorlet is morphologically similar to a carcinoid tumor but measures less than 5 mm and extends beyond the airway basement membrane (3). Pulmonary carcinoid tumorlets are often incidental findings in lung tissue resected for another condition and may occur with chronic inflammation, fibrosis, or bronchiectasis, although they can also occur without overt background lung disease (4). On computed tomography (CT), tumorlets may correspond to small nodules, but imaging findings are nonspecific (5). The coexistence of pulmonary carcinoid tumorlets and invasive lung adenocarcinoma has been reported infrequently (4, 6, 7). Here, we report the case of a patient with spatially separate left upper lobe adenocarcinoma and left lower lobe pulmonary carcinoid tumorlets.

## Case presentation

### Chief complaints

A 71-year-old woman was admitted to our hospital for further evaluation of a newly detected lesion in the apicoposterior segment of the left upper lobe in the setting of multiple bilateral pulmonary micronodules. She reported occasional chest tightness and intermittent cough with sputum but denied hemoptysis, dyspnea, and fever. She reported occasional diarrhea without flushing or other documented features of a functional neuroendocrine syndrome. Serum chromogranin A and 24-hour urinary 5-HIAA were not measured because pulmonary neuroendocrine proliferation was not suspected preoperatively and, in the absence of convincing clinical features of carcinoid syndrome, these tests were not included in the routine preoperative evaluation.

### Medical history

Approximately 2 years before admission, multiple micronodules were incidentally detected in the left lower lobe and right lung on routine chest CT performed as part of a workplace health examination, with each lesion measuring less than 5 mm in maximum diameter. Follow-up chest CT was performed every 6 months over the subsequent 2 years and showed no change in their size or number. No specific treatment was administered during this period. A new lesion in the apicoposterior segment of the left upper lobe was identified 34 days before admission. Bronchoscopy with blind brushing of the involved segment 32 days before admission showed no endobronchial lesion, and brush cytology was negative for malignant cells. The patient had a history of hypertension, coronary heart disease, and diabetes mellitus, all of which were described as well controlled. She had no history of smoking or alcohol use and no family history of lung disease or cancer.

### Physical examination

The available record describes the physical examination as unremarkable, with no documented abnormal cardiopulmonary findings.

### Laboratory examinations

Serum tumor-marker values were within normal limits, including squamous cell carcinoma antigen at 0.29 ng/mL (reference, <2.7 ng/mL), carcinoembryonic antigen at 3.0 ng/mL (0–5.0 ng/mL), cytokeratin 19 fragment at 2.13 ng/mL (0–3.3 ng/mL), and neuron-specific enolase at 10 ng/mL (0–16.3 ng/mL). No clinically significant abnormalities were reported in the preoperative assessment. These serum markers were obtained as part of the routine preoperative evaluation and were not used to establish the histopathologic diagnosis; their normal values did not exclude malignancy. Preoperative complete blood count, liver and renal function tests, and coagulation parameters were all within the normal range. Arterial blood gas analysis showed a partial pressure of oxygen of 70.6 mmHg (reference, 60–100 mmHg), a partial pressure of carbon dioxide of 43.4 mmHg (35–45 mmHg), and a pH of 7.395 (7.35–7.45). Echocardiography revealed impaired left ventricular diastolic function.

### Imaging examinations

Contrast-enhanced thin-section chest CT with 1-mm high-resolution reconstructions showed a lesion in the apicoposterior segment of the left upper lobe that was considered highly suspicious for primary lung cancer, along with multiple micronodules measuring up to 3 mm in the left lower lobe and right lung (**Figures 1A–F**). Brain magnetic resonance imaging, bone scintigraphy, and abdominal CT showed no evidence of distant metastasis. PET/CT was not performed because the patient’s family declined the examination owing to cost considerations. ^68Ga-DOTATOC PET/CT was not pursued because pulmonary neuroendocrine proliferation was not suspected preoperatively.

**Figure 1:**
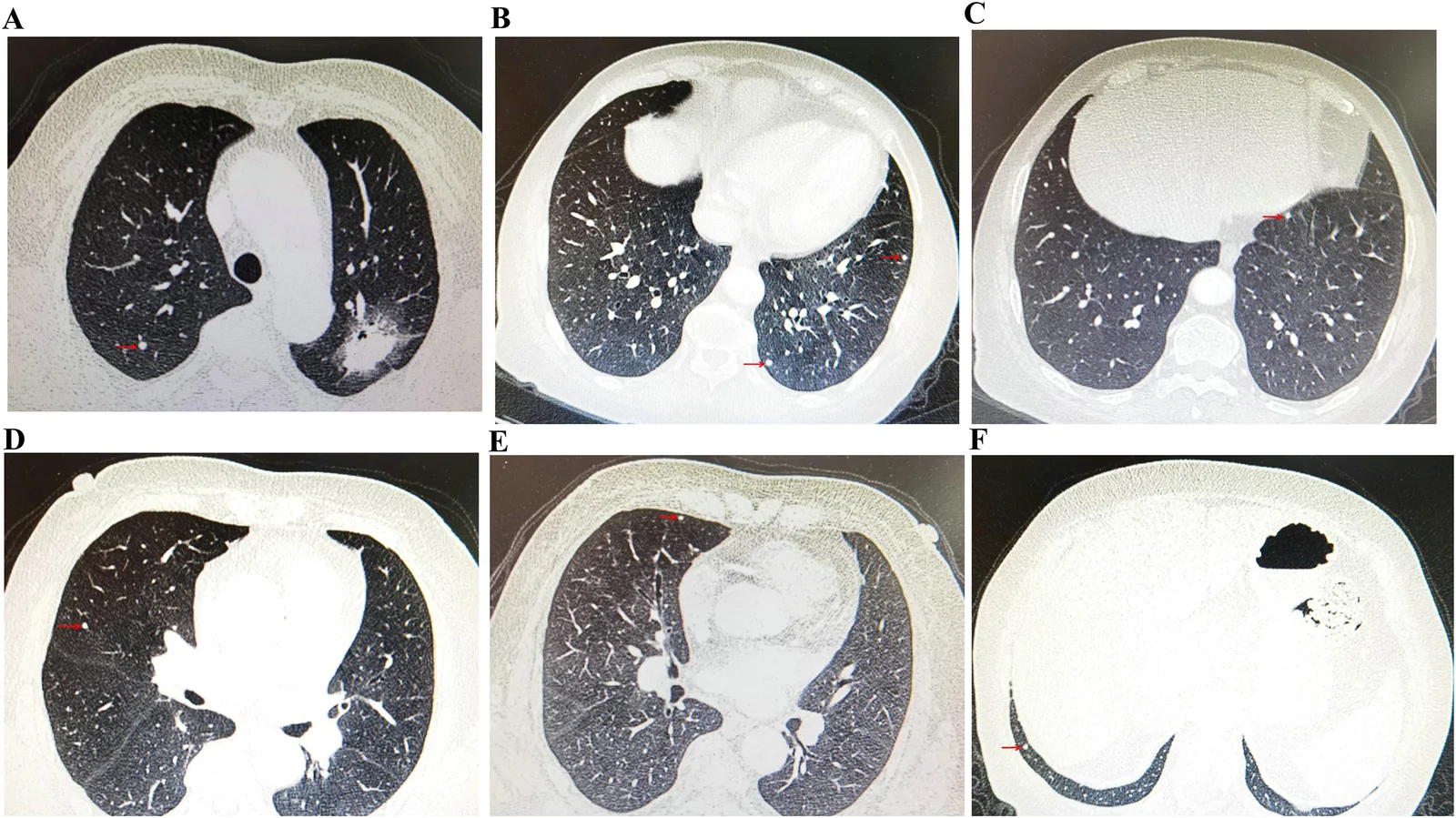
Contrast-enhanced thin-section chest computed tomography with 1-mm high-resolution reconstructions showing the dominant left upper lobe lesion and multiple bilateral micronodules. **(A)** Left upper lobe lesion with a right upper lobe micronodule; **(B, C)** left lower lobe micronodules; **(D, E)** right middle lobe micronodules; and **(F)** right lower lobe micronodule. Red arrows indicate representative micronodules.

### Diagnosis and treatment

Because the patient’s family declined CT-guided biopsy of the left upper lobe lesion owing to concerns about procedural risks, the operative plan was to perform video-assisted thoracoscopic wedge resection with intraoperative frozen-section examination, followed by lobectomy if malignancy was confirmed. Preoperative CT suggested that the multiple left lower lobe micronodules were unlikely to represent metastases. After preoperative discussion with the patient’s family, limited wedge resection was planned only if superficial nodules could be localized by intraoperative palpation, thereby minimizing the loss of normal lung parenchyma. No preoperative CT-guided localization was performed. Intraoperatively, frozen-section examination of the left upper lobe lesion indicated adenocarcinoma, and the procedure was extended to left upper lobectomy with systematic lymph-node dissection. Two superficial left lower lobe nodules were successfully identified by palpation and removed with only a small amount of surrounding lung tissue. Frozen-section examination revealed atypical cells.

Postoperative pathological examination of the left upper lobe nodule revealed a 3.0 × 3.0 × 2.5 cm invasive adenocarcinoma comprising approximately 80% acinar and 20% lepidic patterns (**Figure 2A**). No definite vascular tumor thrombus or visceral pleural invasion was identified. Spread through air spaces was not assessed in the original pathological examination. The dissected lymph nodes were negative for metastasis: station 4L (0/1), station 5 (0/2), station 7 (0/7), station 9 (0/2), station 10 (0/1), station 11 (0/1), station 12 (0/2), and station 13 (0/1). Immunohistochemical analysis showed negative staining for ALK (VENTANA D5F3), HER2 (score 0), ROS1 (score 0), and BRAF, whereas c-Met expression was observed in 60% of tumor cells at moderate intensity. The pathological stage was determined to be pT1cN0M0 (stage IA3). Genetic testing identified an *EGFR* exon 21 L858R mutation, and PD-L1 testing showed a negative tumor proportion score.

**Figure 2:**
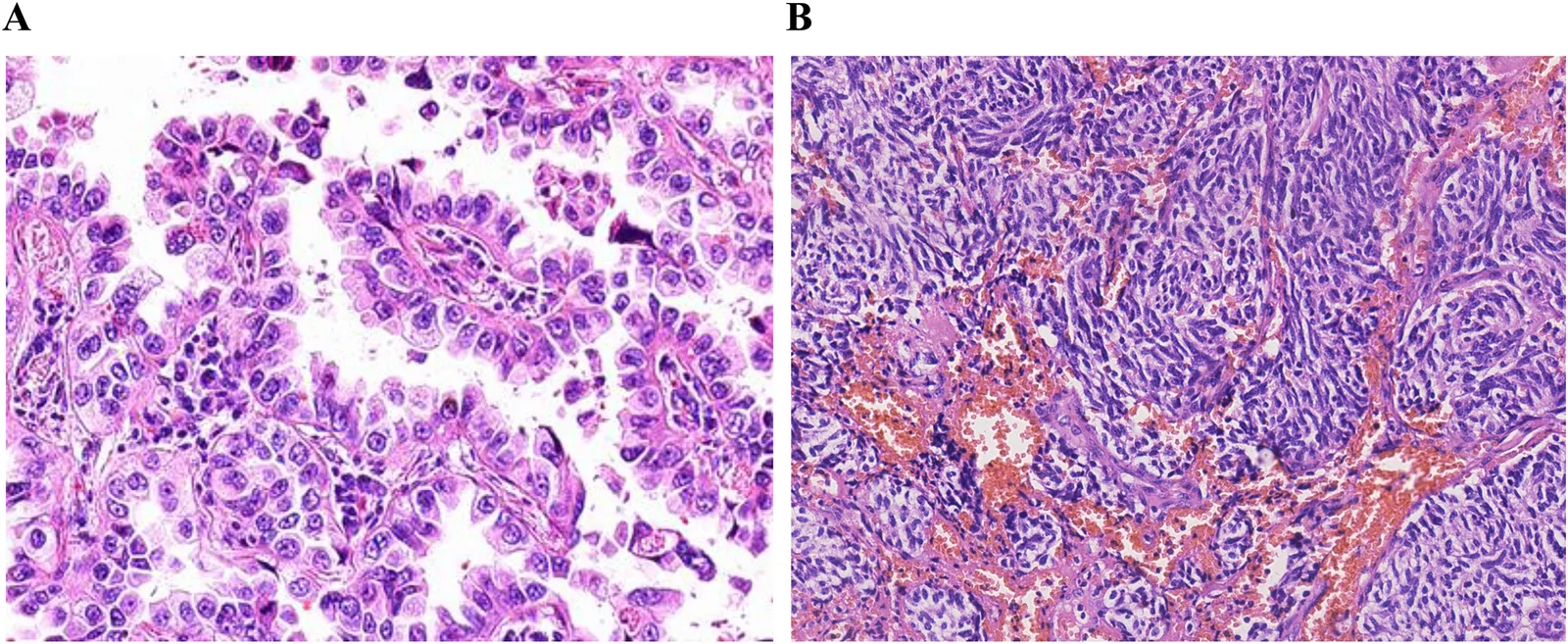
Histopathologic findings. **(A)** Invasive adenocarcinoma of the left upper lobe with gland-forming/acinar and lepidic features (hematoxylin and eosin staining; original magnification ×200). **(B)** Pulmonary carcinoid tumorlet in the left lower lobe, composed of relatively uniform cells arranged in nests (hematoxylin and eosin staining; original magnification ×200).

The left lower lobe wedge-resection specimen measured 2.0 × 0.7 × 0.4 cm and contained multiple pulmonary carcinoid tumorlets (**Figure 2B**). The individual lesions met the microscopic size criterion for tumorlets (<5 mm), although their exact individual dimensions were not recorded. Immunohistochemical analysis showed positivity for AE1/AE3, synaptophysin (Syn), chromogranin A (CgA), CD56, and TTF-1 and negativity for leukocyte common antigen and P40. The Ki-67 proliferation index was approximately 2%, determined by counting at least 500 tumor cells in hotspot areas. Genetic testing of the carcinoid tumorlets did not detect the *EGFR* exon 21 L858R mutation identified in the left upper lobe adenocarcinoma. No adjuvant chemotherapy or osimertinib was administered, and the patient underwent a surveillance program.

### Outcome and follow-up

The postoperative course was uneventful. The chest tube was removed on postoperative day 4, and the patient was discharged on postoperative day 7. At the latest follow-up, the patient remained in good health and underwent surveillance every 6 months at another hospital. All follow-up examinations were reportedly normal, with no evidence of recurrence.

## Discussion

The distinctive feature of this case is the coexistence of a resected left upper lobe adenocarcinoma and multiple pathologically confirmed pulmonary carcinoid tumorlets in the left lower lobe. These spatially separate neuroendocrine lesions fulfilled the diagnostic criteria for tumorlets rather than carcinoid tumors and therefore did not represent a second primary lung malignancy. This unusual coexistence highlights the importance of histologic evaluation of separate pulmonary nodules in patients with lung adenocarcinoma.

The morphology and immunophenotype of the left lower lobe lesions supported pulmonary neuroendocrine differentiation. The lesions consisted of small nests or nodules of relatively uniform cells and were positive for AE1/AE3, Syn, CgA, CD56, and TTF-1, with a Ki-67 proliferation index of approximately 2%. TTF-1 expression was compatible with pulmonary origin, whereas cytokeratin positivity supported epithelial differentiation, and the expression of Syn, CgA, and CD56 supported neuroendocrine differentiation (3, 8). However, immunohistochemistry alone cannot distinguish a tumorlet from a carcinoid tumor; this distinction requires assessment of lesion size and invasion beyond the basement membrane. If classification of a carcinoid tumor as typical or atypical is required, mitotic activity and necrosis, rather than the Ki-67 index alone, remain the principal criteria (1, 9).

Pulmonary tumorlets are usually detected incidentally in lung specimens resected for other diseases and are frequently associated with chronic inflammation, fibrosis, or bronchiectasis, although they may also occur without overt background lung disease (4). Pulmonary tumorlets may appear on CT as subcentimeter nodules, but their imaging features are nonspecific and do not permit a definitive diagnosis without histopathologic examination (5). Multiple tumorlets may consequently mimic intrapulmonary metastatic disease (10). In this patient, only the left lower lobe nodules were histologically examined. The residual right-lung micronodules may represent additional tumorlets, scars, intrapulmonary lymph nodes, other benign nodules, or separate neoplasms and should not be designated as tumorlets without tissue confirmation. Their reported stability for approximately 2 years favors an indolent process but does not establish their histologic identity. Continued radiologic surveillance is therefore warranted, with further evaluation or tissue sampling if interval growth or suspicious morphologic changes occur.

Pulmonary carcinoid tumorlets have previously been reported as incidental findings in lung specimens resected for adenocarcinoma (4). The present case is distinct from reports of adenocarcinoma coexisting with a true carcinoid tumor because the neuroendocrine lesions were located in a different lobe and fulfilled the size criteria for multiple tumorlets (11). They therefore do not constitute a second carcinoid tumor and should not be staged as synchronous double primary lung cancers. Pulmonary carcinoid neoplasms may also present as bilateral metachronous lesions of differing histologic grades, illustrating the broader diagnostic complexity of multifocal neuroendocrine lesions (12).

The detection of *EGFR* L858R in the adenocarcinoma but not in the tumorlets is biologically plausible and compatible with separate origins. Activating EGFR mutations are established drivers in a subset of lung adenocarcinomas, whereas well-differentiated pulmonary neuroendocrine proliferations typically have a distinct molecular landscape and rarely harbor NSCLC-type driver alterations (13, 14). Nevertheless, such discordance cannot independently establish clonality, because a negative result in the minute tumorlets may be affected by limited lesional tissue or assay sensitivity (15). Their spatial separation and distinct histologic phenotypes provide stronger evidence that the adenocarcinoma and tumorlets represent separate pathologic processes.

The 3.0-cm, node-negative adenocarcinoma was classified as pT1cN0M0, stage IA3, according to the ninth-edition TNM classification (16). Following complete resection, the omission of adjuvant chemotherapy is consistent with recommendations that do not support routine adjuvant chemotherapy for stage IA disease (17). Although the ADAURA trial demonstrated an overall survival benefit with adjuvant osimertinib in patients with completely resected, *EGFR*-mutated stage IB–IIIA NSCLC, as classified according to the seventh edition of the TNM staging system, patients with stage IA disease were not included (18). The benefit of adjuvant osimertinib in stage IA2–IA3 disease is currently being evaluated in the ADAURA2 trial (19). Consequently, its clinical benefit in stage IA3 disease has not yet been established, and routine use in this setting cannot be supported by ADAURA evidence alone. The *EGFR* result may nevertheless inform treatment selection if recurrent or metastatic disease subsequently develops.

Multiple tumorlets should raise consideration of DIPNECH; however, neuroendocrine cell hyperplasia and tumorlets may also occur as secondary/reactive changes in association with other pulmonary diseases or neoplasms, and therefore should be interpreted in the appropriate clinicopathologic and radiologic context (13). DIPNECH syndrome is generally supported by chronic respiratory symptoms, airflow obstruction, multiple bilateral pulmonary nodules, mosaic attenuation or air trapping on CT, and constrictive bronchiolitis (20). Although the patient reported occasional chest tightness and intermittent cough with sputum, the available records did not document chronic progressive respiratory symptoms, wheezing, or dyspnea. In this patient, preoperative pulmonary function testing showed no obvious abnormality; the available CT reports did not describe mosaic attenuation or air trapping; and the pathological report did not document diffuse neuroendocrine cell hyperplasia in the background lung or constrictive bronchiolitis. Therefore, the available clinicoradiological and pathological findings were insufficient to establish either pathological DIPNECH or DIPNECH syndrome. Nevertheless, a DIPNECH-spectrum process cannot be completely excluded because dedicated expiratory CT and systematic reassessment of the background lung were unavailable.

Overall, this case emphasizes the importance of histologic assessment of spatially separate nodules in patients with lung adenocarcinoma, because tumorlets may mimic intrapulmonary metastases. Unsampled nodules could remain radiologically indeterminate and undergo longitudinal surveillance rather than being presumed to represent either metastases or additional tumorlets.

## Data Availability

Data are not publicly available because they contain information that could compromise patient privacy but may be available from the corresponding author subject to ethical and institutional requirements.
